# Stemness-Relevant Gene Signature for Chemotherapeutic Response and Prognosis Prediction in Ovarian Cancer

**DOI:** 10.1155/sci/2505812

**Published:** 2025-03-27

**Authors:** Kaixia Zhou, Xiaolu Ma, Tianqing Yan, Hui Zheng, Suhong Xie, Lin Guo, Renquan Lu

**Affiliations:** ^1^Department of Clinical Laboratory, Fudan University Shanghai Cancer Center, Shanghai 200032, China; ^2^Department of Oncology, Shanghai Medical College, Fudan University, Shanghai 200032, China

**Keywords:** bioinformatics, cancer stem cells, chemotherapy, ovarian cancer, prognostic model

## Abstract

**Background:** Ovarian cancer (OC) stands as the leading cause of cancer-related deaths among women, globally, owing to metastasis and acquired chemoresistance. Cancer stem cells (CSCs) are accountable for tumor initiation and exhibit resistance to chemotherapy and radiotherapy. Identifying stemness-related biomarkers that can aid in the stratification of risk and the response to chemotherapy for OC is feasible and critical.

**Methods:** Gene expression and clinical data of patients with OC were downloaded from The Cancer Genome Atlas (TCGA) and the Gene Expression Omnibus (GEO) database. Four thousand three hundred seventeen stemness-related genes (SRGs) were acquired from the StemChecker database. TCGA was used as the training dataset, while GSE30161 served as validation dataset. Univariate Cox regression analysis was used to identify overall survival (OS)-related SRGs, and multivariate Cox regression analysis and random survival forest analysis were used for generating stemness-relevant prognostic model. Kaplan–Meier plots were used to visualize survival functions. Receiver operating characteristic (ROC) curves were used to assess the prognostic predictive ability of SRG–based features. Associations between signature score, tumor immune phenotype, and response to chemotherapy were analyzed via TIMER 2.0 and oncoPredict R package, respectively. A cohort of Shanghai Cancer Center was employed to verify the predictive robustness of the signature with respect to chemotherapy response.

**Results:** Seven SRGs (actin-binding Rho activating C-terminal like (ABRACL), growth factor receptor bound protein 7 (GRB7), Lin-28 homolog B (LIN28B), lipolysis stimulated lipoprotein receptor (LSR), neuromedin U (NMU), Solute Carrier Family 4 Member 11 (SLC4A11), and thymocyte selection associated family member 2 (THEMIS2)) were found to have excellent predictive potential for patient survival. Patients in the high stemness risk group presented a poorer prognosis (*p*  < 0.0001), and patients with lower stemness scores were more likely to benefit from chemotherapy. Several tumorigenesis pathways, such as mitotic spindle and glycolysis, were enriched in the high stemness risk group. Tumor with high-risk scores tended to be in a status of relatively high tumor infiltration of CD4+ T cells, neutrophils, and macrophages, while tumor with low-risk scores tended to be in a status of relatively high tumor infiltration of CD8+ T cells.

**Conclusions:** The stemness-relevant prognostic gene signature has the potential to serve as a clinically helpful biomarker for guiding the management of OC patients.

## 1. Introduction

According to GLOBOCAN estimates, approximately 314,000 women were diagnosed with ovarian cancer (OC) in 2020, resulting in 20,700 deaths. The morbidity and mortality of patients with OC are both estimated to rank eighth among women worldwide, posing a serious threat to women's health [1]. Over the last three decades, there have been advancements in diagnostic methods and treatment options for OC, leading to some notable progress. However, OC remains a significant medical challenge, and the 5-year survival rate has seen limited improvement. This is attributed to the lack of effective screening tools for OC, with over half of patients facing recurrence within 2 years after treatment.

Mounting evidence has demonstrated that OC treatment failures, such as relapse and metastasis, are rooted in a small population of cancer stem cells (CSCs), which possess the ability to manipulate various signaling pathways and initiate the formation of new tumors [2]. CSCs were identified for the first time by Bonnet and Dick [3] in 1997, who illustrated that a single leukemic cell exhibited stem cell-like properties, including self-renewal and cell differentiation abilities, and could initiate a condition similar to acute myeloid leukemia when transplanted into nonobese diabetic/severe combined immune-deficient mice as recipients. Further studies on many types of solid tumors, including brain, breast, liver, lung, pancreas, or ovary, yielded similar findings [4]. Therefore, the development of specific therapies targeting CSCs has been considered a promising avenue to improve the outcomes of cancer patients. Throughout the years, cancer research has focused on understanding the biological attributes of CSCs, delineating the characteristics of this small subpopulation, and formulating novel strategies for their identification and targeting so as to effectively combat drug resistance, relapse, and metastasis effectively.

Two pivotal properties of CSCs are their ability to self-renew and their state of quiescence. Aberrant self-renewal in CSCs, stemming from the dysregulation of signal transduction processes, confers a high level of proliferative activity. The main pathways implicated in this phenomenon include Hedgehog, Notch, Wnt/*β*-catenin, HMGA2, Bcl-2, Bmi-1, and PI3K/AKT/mTOR pathways. These observations indicated that these pathways are pertinent to the drug resistance of these cells [5]. In OC, targeting Notch could sensitize tumors to platinum therapy by depleting CSCs [6]. Additionally, calcium channel blockers disrupted the stemness in ovarian CSCs, and the combinatorial usage of manidipine and paclitaxel showed a synergistic effect in xenograft mouse models [7].

Immune evasion has been proposed to be a fundamental feature of CSCs [8], as these cells have been proven to be capable of achieving remarkable immune resistance, and thereby, contribute to cancer metastasis and relapse. On the one hand, CSCs orchestrate crucial crosstalk with infiltrating tumor-associated immune and stromal cells within tumors, driving them to adopt pro-tumoral phenotypes and contributing to therapeutic resistance. On the other hand, CSCs seem to exhibit higher levels of inhibitory checkpoint receptors compared to non-CSCs, thereby, dampen immune cells' antitumor activity [9]. For example, CSCs isolated from various cancer, such as breast CSCs and colon CSCs, can evade immune surveillance through overexpression of PD-L1 [10].

In this study, using stemness-related genes (SRGs) extracted from 26 publicly available stemness gene sets, along with differentially expressed genes between The Cancer Genome Atlas (TCGA) and GTEx for OC, we employed univariate Cox regression analysis to identify candidate SRGs strongly associated with the prognosis. The stemness-risk model was then constructed through a combination of Cox regression and random survival forest analyses. Furthermore, we delved into its connections with the prognosis of OC, TME patterns, molecular functions, and the effectiveness of chemotherapy. In conclusion, we developed a stemness-risk score to delineate the stemness profiles, which demonstrated strong predictive capabilities for both prognosis and chemotherapy response in patients with OC.

## 2. Materials and Methods

### 2.1. OC Datasets Acquisition

In this study, we analyzed 584 OC samples with matched clinical and survival data from two independent datasets: the TCGA-OV cohort and the GSE30161 cohort. Regular ovarian tissue samples were obtained from GTEx and deemed to be normal controls. A SRG set was consisted of common genes retrieved from 26 stemness gene sets downloaded from StemChecker (http://stemchecker.sysbiolab.eu/), which was based on updated collection of published stemness signatures defined by RNAi screens, gene expression profiles, and so on. The expression profiling data of OC cell lines derived from CCLE data, which is hosted on the Cancer Dependency Portal (Demap, https://depmap.org/portal/), where it has been harmonized with corresponding genomics and sequencing data.

### 2.2. Patients and Clinical Specimens

OC tissues were collected at Fudan University Shanghai Cancer Center from October 27, 2015 to July 7, 2023 during surgical resection in 30 patients, who were used as a real-world cohort. The samples were preserved in RNA Stabilization Reagent for Animal Tissue (CWBIO, CWY042) and stored in liquid nitrogen until RNA extraction was performed. Patients who experienced recurrence within 6 months after chemotherapy were classified as chemotherapy resistant, while those who remained recurrence-free for over 6 months were considered chemotherapy sensitive. The pathological stage was determined based on the AJCC and TNM classification system, and specific details can be found in Table 1. Informed consent was signed by all patients. The research was approved by the Ethics Committee of Fudan University Shanghai Cancer Center and carried out in accordance with the ethical standards as formulated in the Helsinki Declaration.

### 2.3. Developing the Prognostic Stemness-Associated Gene Signature

The Combat function from the sva R package (v 3.46.0) was used to remove batch effects from the public datasets. The Wilcoxon test was used to identify SRGs that were differentially expressed between ovarian tissue from the GTEx dataset and tumor tissues from the TCGA dataset (|log_2_ FC| > 1, FDR < 0.05). Then, in the TCGA cohort, univariate Cox regression analysis was used to screen SRGs that were predictive of prognosis. Next, we established a random survival forest model for model reduction via the randomForestSRC R package (v 3.2.1). Genes with relative importance >0.5 were selected in that random forest, where the relative significance of each variable would be quantified robustly. Ultimately, the gene combination that produced the best log-rank *p* value for the stemness-risk model construction was found: risk score = ∑(*βi* × Exp *i*), where *βi* is the coefficient of the corresponding gene and Exp *i* was the expression value of the gene. Every patient's risk score was determined using the signature basis, and the median split was used to classify all patients into high- or low-risk groups. The log-rank test was used to compare the Kaplan–Meier survival curves of these two groups. Using the timeROC R package (v 0.4), a time-dependent receiver operating characteristic (ROC) analysis was performed to evaluate the predictive accuracy of the signature.

### 2.4. Chemotherapy Response Predictions

In order to predict chemical sensitivity, the half-maximal inhibitory concentration (IC_50_) values of multiple drugs (Cisplatin, Niraparib, Olaparib, and Paclitaxel) in each organic sample were calculated using the oncoPredict R package (v 0.2). This package was developed based on the Genomics of Drug Sensitivity in Cancer (GDSC) and employed ridge regression.

### 2.5. Molecular Characterization

To investigate distinct molecular and signaling pathways between the high- and low-stemness risk groups, we conducted gene set variation analysis using hallmark gene sets obtained from the Molecular Signatures Database (MSigDB). |*t*-values| ≥ 2 were considered significantly enriched.

### 2.6. Statistical Analysis

Data are presented as means ± SD or as boxplots, and all statistical analyses were conducted in the GraphPad Prism 9.0 and R software (v 4.2.2). Statistical analysis was performed using the unpaired student's *t*-test and the Pearson chi-square test as appropriate for the dataset. The Kaplan–Meier method and log-rank Mantel–Cox test were used for survival analysis. Functional data are representative of at least triplicates, unless otherwise specified. A two-tailed *p* value <0.05 was regarded as statistically significant.

### 2.7. Real-Time PCR

As directed by the manufacturer, TRIzol (Thermo Fisher, 15596026) was used to extract total RNA. The PrimeScriptTM RT Master Mix (Takara, RR036A) was used to synthesis cDNA. TB Green Premix Ex Taq II (Takara, RR420A) was used for the RT-PCR, which was carried out on an ABI PRISM Detection System (Applied Biosystems/Life Technologies). The data was analyzed using the 2^−*ΔΔ*CT^ method, with GAPDH acting as a loading control. Table S4 contains primer sequences.

### 2.8. Cell Culture and Small Interfering RNA (siRNA) Transfection

The human OC cell line OVCAR8 was purchased from Life Technologies and cultivated in Roswell Park Memorial Institute (RPMI) 1640 medium (cytiva, SH30809.01) supplemented with 1% penicillin–streptomycin (Gibco, 15140122) and 10% fetal bovine serum (Yeasen, 40130ES76). Each culture was kept at 37°C in an incubator with 5% CO_2_. GenePharma (Shanghai, China) synthesized siRNA against thymocyte selection associated family member 2 (THEMIS2; si-THEMIS2) and the negative control (NC), and the sequences of THEMIS2 (*Homo sapiens*) siRNA were 5'-UGGAGCCGGUGCCGCUGCATT-3', 5'-UCAUGUCGACCCACAGGAUTT-3', and 5'-AGGAUCCAGCCCUGAAAGATT-3'. JetPRIME transfection reagent (Polyplus, 114-15) was used to transfect OC cells grown in six-well plates at approximately 75% confluence, as per the manufacturer's instructions.

### 2.9. Western Blotting

After a cold PBS wash, OC cell lines were lysed for 10 min on ice in RIPA lysis buffer (Beyotime, P0013C) supplemented with PMSF (Beyotime, ST506) and a protease and phosphatase inhibitor cocktail (Beyotime, P1048). The protein lysate was then centrifuged for 15 min at 4°C, and the supernatant was gathered. Using 5× SDS loading buffer (Beyotime, P0015L), protein supernatants were made and they were denatured for 5 min at 100°C. Protein samples were electroblotted onto PVDF membranes (Merck Millipore, ISEQ00010) after separation by PAGE gel electrophoresis. After 15 min of room temperature incubation in QuickBlockTM Western (Beyotime, P0252), the membranes were incubated with primary antibodies against GAPDH (Proteintech, 60004-1), THEMIS2 (NovoPro, 162595), c-Myc (Santa Cruz, sc-40), Nanog (Affinity Biosciences, AF5388), and OCT4 (NovoPro, 104487) for an additional night. Primary antibody dilution buffer (Beyotime, P0023A) was used to dilute primary antibodies. Following TBST washing, the membranes were incubated for 1 h at room temperature with either rabbit secondary antibodies (Beyotime, A0208) or goat anti-mouse HRP-conjugated antibodies (Beyotime, A0192).

### 2.10. Sphere-Formation Assay

OVCAR8 cells were processed into single cells and seeded at a density of 1000 cells per well in six-well ultralow attachment plates (Corning) containing sphere formation medium. The medium consisted of RPMI-1640 supplemented with 10 ng/mL EGF (Proteintech, HZ-1326), 10 ng/mL FGF (Proteintech, HZ-1285), and 1× B-27 supplement (Invitrogen, 17504044). The spheres were allowed to form over 7 days in humidified incubator at 37°C with 5% CO_2_. At the end point, the diameter of spheres was assessed under a brightfield microscope.

## 3. Result

### 3.1. Development of a Prognostic SRG Signature

**Figure 1:**
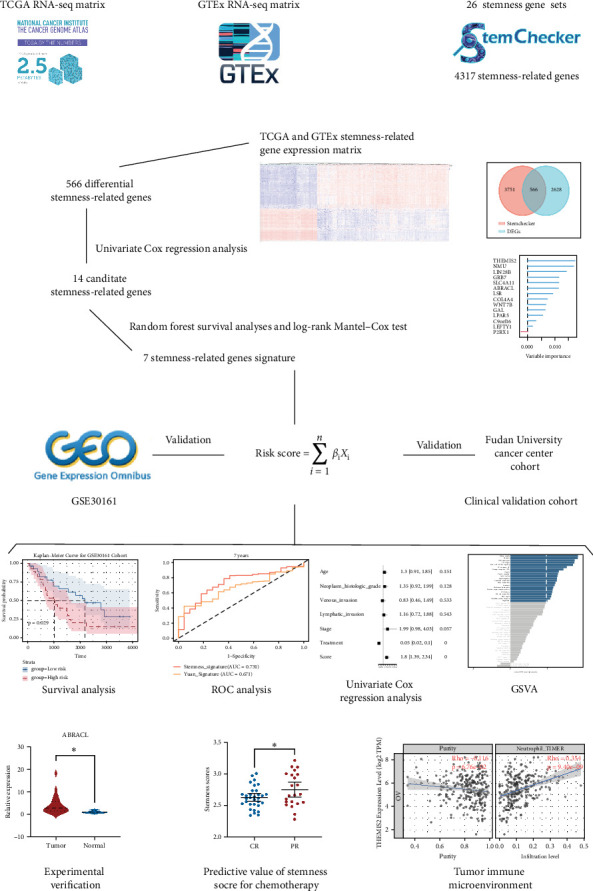
Work flow of this study.

The overall scheme of the construction of this study is displayed in Figure 1 and the OC patient's clinical and demographic characteristics in all the cohorts are summarized in Table 2. By combining genes from 26 stemness gene sets derived from StemChecker, we have obtained 4317 SRGs. A total of 566 of these genes showed distinct levels of expression in ovarian tissue in the GTEx dataset and OC patients in the TCGA dataset (Figure S1A–C). Performing a univariate Cox regression analysis on the TCGA cohort, we found that 14 of the aforementioned potential genes had a significant association with OS. Table S1 furnishes a comprehensive account along with the coefficients of these genes. Next, genes with very little significance for OS were filtered out using random forest survival analysis, and seven genes (actin-binding Rho activating C-terminal like (ABRACL), growth factor receptor bound protein 7 (GRB7), Lin-28 homolog B (LIN28B), lipolysis stimulated lipoprotein receptor (LSR), neuromedin U (NMU), Solute Carrier Family 4 Member 11 (SLC4A11), and THEMIS2) with relative importance >0.5 were screened (Figure S1D). The seven genes were then put together into 127 (2^7^ − 1) alignment assemblies, and the log-rank Mantel–Cox test was used to assess prognostic models. As Figure S1E shows the stemness-risk model construction process involved extracting the top-rank signature, which consisted of seven genes (ABRACL, GRB7, LIN28B, LSR, NMU, SLC4A11, and THEMIS2), from the −log_10_ (log-rank *p*) values of the top 10 ranking models. The total of these seven coefficient-weighted genes' expression levels was used to define the risk score: Risk score = (0.1837873 × ABRACL) + (0.0688455 × GRB7) + (0.0860518 × LIN28B) + (0.0727462 × LSR) + (0.0871861 × NMU) + (0.1362820 × SLC4A11) + (0.0008634 × THEMIS2). Further examination delved into the differences in expression patterns of these genes between ovarian tissues in GTEx and OC tissues in the TCGA dataset. Compared to tumor tissues, normal tissues had lower levels of these genes' mRNA (Figure S2), indicating that they may play different roles in OC progression.

### 3.2. The Risk-Stratification Value of the Signature in OC

**Figure 2:**
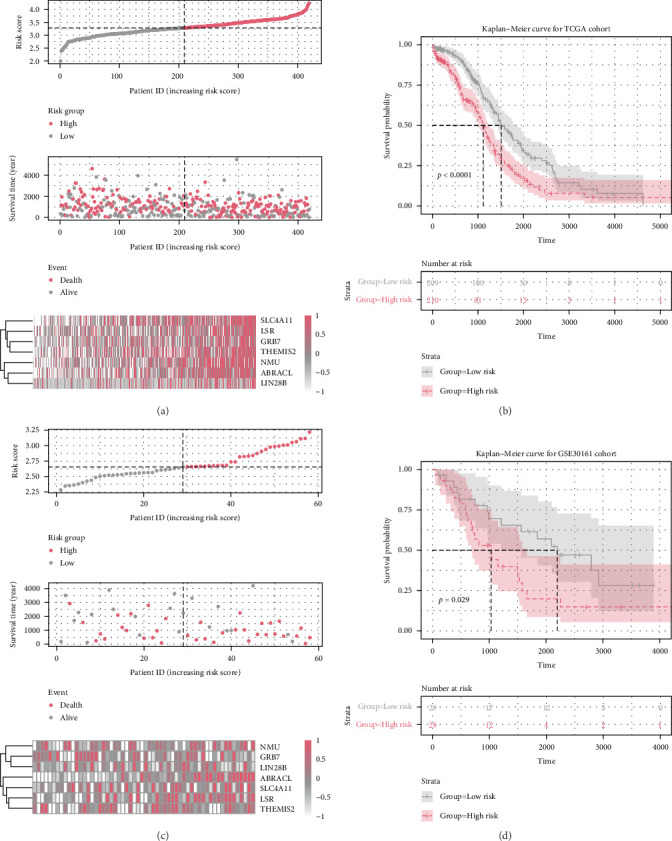
Validation of the prognosis signature for stemness-related genes (SRGs). (A, C) The risk score and expression of selected seven SRGs in the ovarian cancer (OC) patients in The Cancer Genome Atlas (TCGA) cohort and GSE30161 cohort. Top panel, the low- and high-risk groups defined based on the cutoff value (median). Middle panel, the survival status and duration in the OC cases in the low- and high-risk groups. Bottom panel, heatmap of the expression of the seven SRGs in each tumor. (B, D) Kaplan–Meier overall survival (OS) analysis of OC patients classified according to stemness-relevant prognostic gene signature in TCGA and GSE30161 datasets.

**Figure 3:**
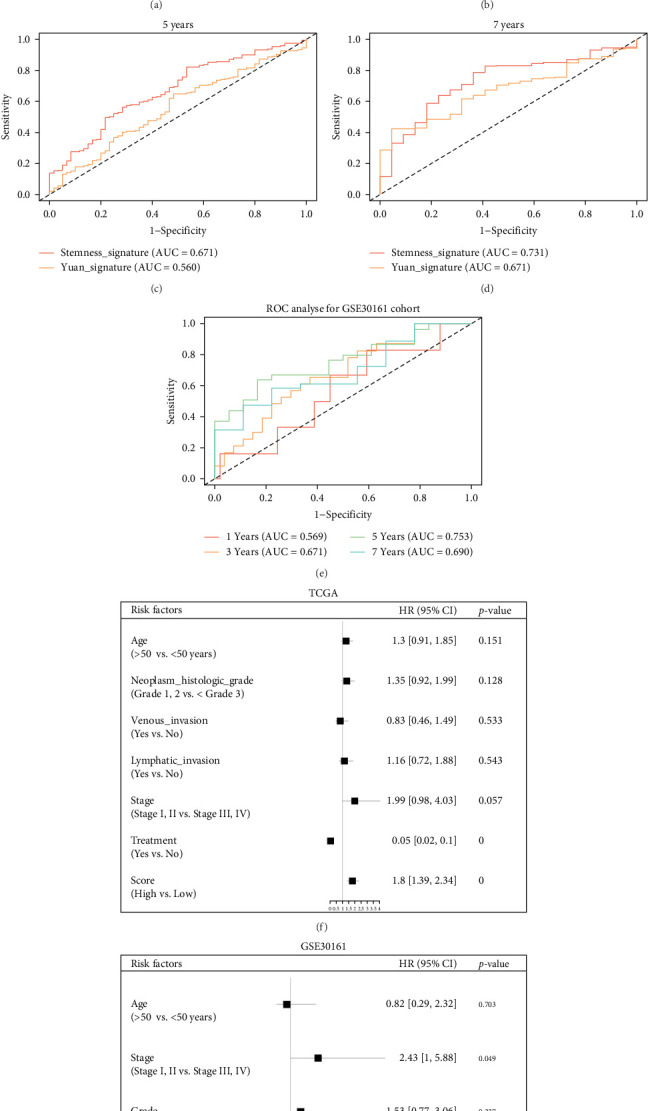
Independent prognostic analysis of risk scores and clinical parameters. (A–D) Time-dependent receiver operating characteristic (ROC) analyses of predictive accuracy of the signature and Yuan signature, a published stemness index prognostic signature, for 1-, 3-, 5-, and 7-year overall survival (OS) of patients in The Cancer Genome Atlas (TCGA) cohort. (E) Time-dependent ROC analyses of predictive accuracy of the signature for 1-, 3-, 5-, and 7-year OS of patients in the GSE30161 cohort. (F, G) Forest plots of univariate and multivariate Cox regression analyses of the OS in the TCGA and GSE30161 cohorts. The square represents HR and the horizontal line represents 95% CI.

Applying this formula, the risk score of each OC patient in the TCGA dataset was computed and the median risk score (3.285) was served as the cut-off point for assigning patients to high-risk and low-risk groups (Figure 2A). The results of Kaplan–Meier analysis revealed a significantly higher overall survival (OS) rate for patients with low-risk scores compared to those with high-risk scores (*p*  < 0.0001; Figure 2B). To confirm the prognostic value of the signature, we further validated the OS predictive ability of the stemness risk model in another cohort, and the Kaplan–Meier survival curves also showed that OC patients with a high stemness risk score had a shorter OS time than those with a low stemness risk score in GSE30161 (log-rank test, *p*=0.029; Figure 2C,D). The accuracy of the signature in predicting OS at 1, 3, 5, and 7 years was then assessed using time-dependent ROC curves, yielding area under the curve (AUC) values of 0.646, 0.635, 0.671, and 0.731, respectively (Figure 3A–D). Based on the stemness index (mRNAsi), Yuan et al. [11] identified 9-mRNA mRNAsi-related signature. Here, we compared the predictive ability of the two models to determine whether our stemness-related seven-gene signature is superior to the Yuan-signature. As Figure 3 shows the ROC curve revealed that the predictive performance of our seven-gene stemness model was significantly better than that of the Yuan model (AUC values: 0.489, 0.570, 0.560, and 0.671, respectively). Besides, the time-dependent ROC curves for the GSE30161 cohort showed AUCs of 0.569 at 1 year, 0.671 at 3 years, 0.753 at 5 years, and 0.690 at 7 years (Figure 3E). Our model obtains a consistent prediction.

Furthermore, we investigated the association between clinicopathological features and the risk score. In all cohorts (TCGA and GSE30161), there was no significant association between the high-risk score and clinicopathological characteristics such as age, histologic grade, stage, venous invasion, and lymphatic invasion (Tables S2 and S3). Univariate and multivariate Cox regression analyses revealed that the risk score (*p*  < 0.001) independently served as a risk factor for OS in OC patients within the TCGA cohort (Figure 3F). Similarly, in the GSE30161 cohort, the risk score (*p*=0.032), tumor stage (*p*=0.049), and suboptimal therapeutic response (*p*=0.007) were identified as significant independent prognostic indicators for OS in patients with OC (Figure 3G). In summary, these findings underscore the stemness and independence of our SRG signature in predicting an unfavorable prognosis in OC patients.

### 3.3. Biological Pathways Associated With the Signature

**Figure 4:**
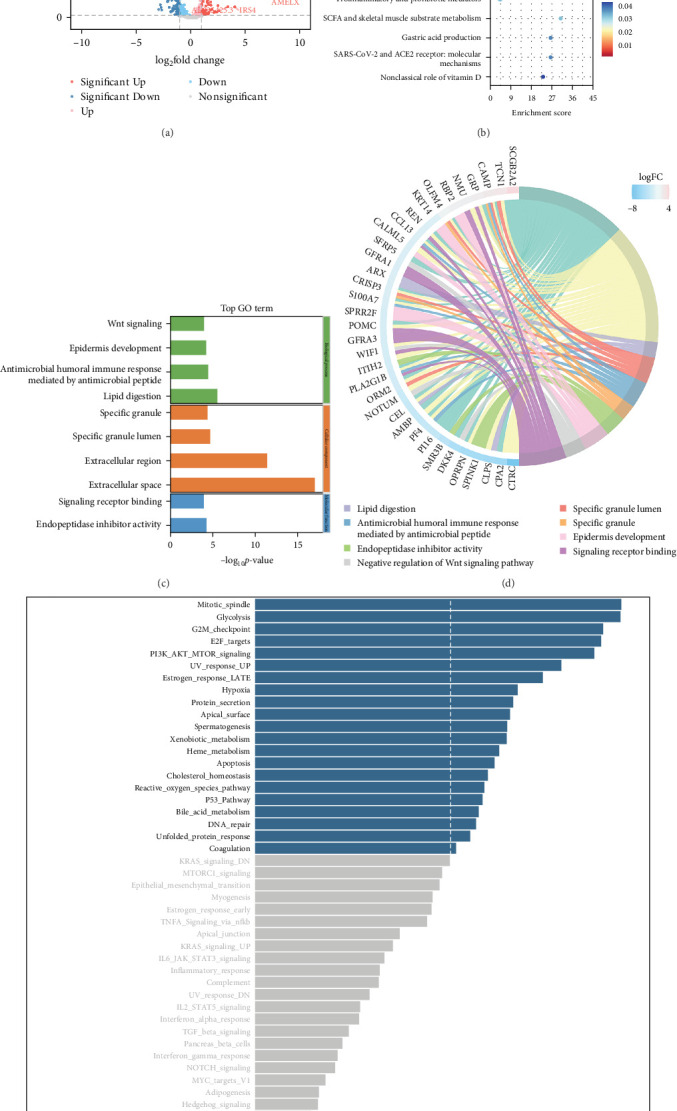
Kyoto Encyclopedia of Genes and Genomes (KEGG) analysis, Gene Ontology (GO) analysis, and Gene set variation analysis (GSVA) based on stemness-associated signature. (A) Volcano map screening for differential genes. (B) Bubble map showing the enriched KEGG pathway. (C, D) GO enrichment analysis. (E) GSVA of differentially enriched hallmark gene sets in the low- and high-risk groups. The vertical dotted lines represent *t*-value = 2. A *t*-value for the GSVA score greater than 2 represented significantly enriched pathways in the high-risk group.

Dysregulation of intracellular signaling contributes to tumor initiation, progression, and therapeutic response to drugs. In this study, to find genes that were expressed differently between the high-risk and low-risk groups, RNA-Seq transcriptomes of OC samples from TCGA were analyzed (Figure 4A). Our investigation revealed a notable enrichment of pathways connected to the Wnt signaling pathway and embryonic stem cell pluripotency pathways in high-risk OC patients (Figure 4B). Additionally, the high-risk group's Gene Ontology (GO) enrichment analysis uncovered an activation of the Wnt signaling pathway, which was significantly overrepresented among the genes that are expressed differently in the high-risk and low-risk groups (Figure 4C,D). Furthermore, we conducted gene set variation analysis to investigate the connection between the stemness-risk score and cellular function. As depicted in Figure 4E, distinct functional signaling pathways were enriched in low- and high-risk groups. More specifically, several tumorigenesis pathways, such as mitotic spindle, glycolysis, G2/M checkpoint, E2F targets, and PI3K AKT mTOR signaling, were more numerous in the high-risk cohort.

### 3.4. TME and Immune Cell Infiltration Associated With the Signature

**Figure 5:**
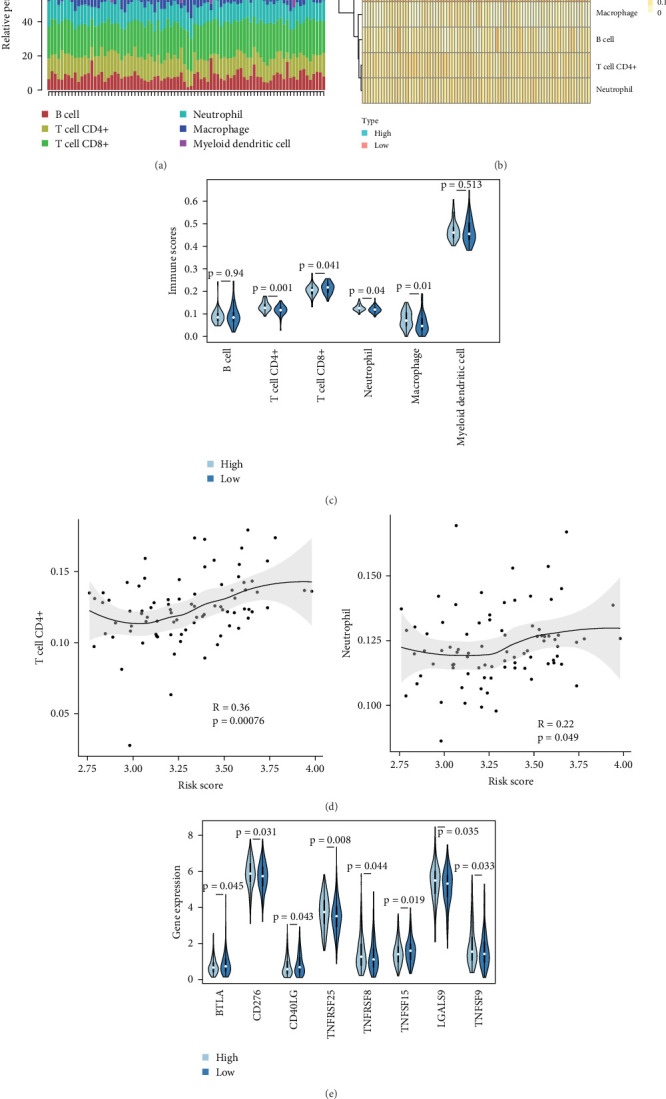
Identify immune landscape of ovarian cancer (OC) in The Cancer Genome Atlas (TCGA) cohort based on stemness-associated signature. (A) Proportion of immune cells in OC tissues. Heatmap (B) and box plot (C) showing the differences of six infiltrating immune cells between low- and high-risk groups. (D) The correlation between the risk score and immune cell types, such as CD4+ T cell (upper panel) and neutrophils (bottom panel), in OC tissues was analyzed. (E) Average expression of immune checkpoint genes (ICGs) in the low- and high-risk groups of OC patients based on stemness-associated signature.

Increasing evidence shows that CSCs interact with components of the TME, such as immune cells and various cytokines, thereby, promoting drug resistance in various cancers [12]. In order to investigate whether alterations in the immune microenvironment are associated with the signature, we uploaded whole transcriptome data of the top 10% low-risk and high-risk patients from the TCGA-OV dataset to TIMER 2.0 (http://timer.cistrome.org/) to determine the abundance of immune cells at various risk levels (Figure 5A). Our results disclosed that tumors with high-risk scores tended to have comparatively high tumor infiltration of neutrophils, macrophages, and CD4+ T cells, while low-risk tumors typically exhibit a comparatively high level of CD8+ T cell tumor infiltration (Figure 5B,C). In addition, our findings, presented in Figure 5D, indicated that the infiltration of CD4+ T cells and neutrophils is highly correlated with risk scores of patients with OC in TCGA cohort. Among the seven SRGs in the signature, the expression levels of ABRACL, GRB7, SLC4A11, and THEMIS2 had a favorable correlation with neutrophils in OC, while the expression levels of GRB7, LIN28B, LSR, and THEMIS2 showed a positive relationship with macrophages (Figure S3). Our findings were consistent with the distribution of stemness-risk scores.

We assessed the expression levels of known immune checkpoint molecules in both low-risk and high-risk groups to determine whether changes in the expression patterns of immune checkpoint genes (ICGs) are linked the signature. In the TCGA cohort, tumors with high-risk score were characterized by relatively high expression of CD276, LGALS9, TNFRSF25, TNFRSF8, and TNFSF9, while individuals with low-risk scores typically exhibited a comparatively high expression of BTLA, CD40LG, and TNFSF15 (Figure 5E). These results again demonstrated that patients with OC in the low- and high-risk categories for stemness have different immune conditions in the tumor microenvironment and confirmed the clinical relevance of our signature.

### 3.5. The Significance of Stemness-Risk Score in Sensitivity Predictions for Chemotherapy

**Figure 6:**
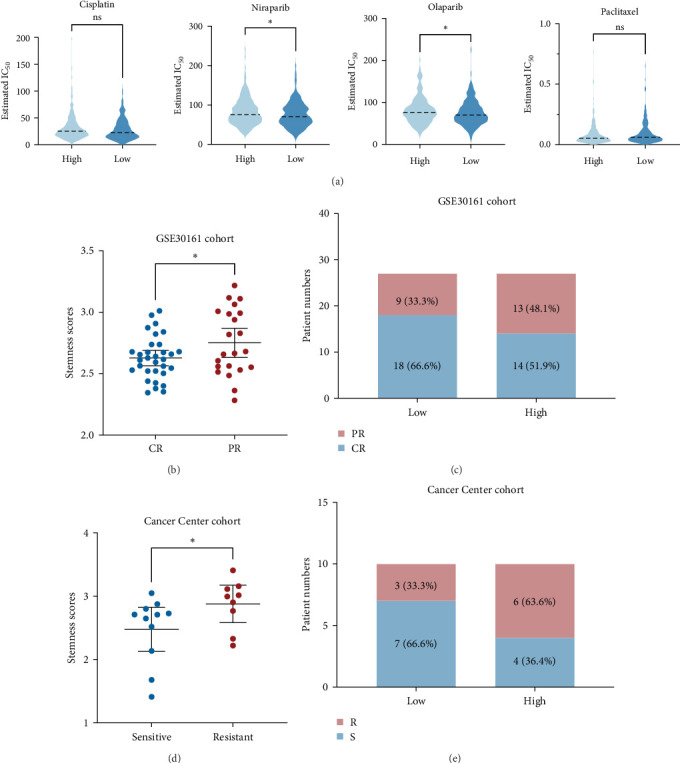
Chemotherapy response prediction. (A) Violin plots for the estimated half-maximal inhibitory concentration (IC_50_) of chemotherapy drugs in high- and low-risk groups. (B) Stemness scores of patients in complete response (CR) and partial response (PR) groups in GSE30161 cohort. (C) Distributions of complete responder and partial responder to chemotherapy in high- and low-risk group. (D) Stemness scores of patients between chemosensitive (S) and chemoresistant (R) groups in Cancer Center cohort. (E) Distributions of responder (S) and nonresponder (R) to chemotherapy in high- and low-risk group.

Currently, cytoreductive surgery and systemic chemotherapy remain the conventional treatment for OC patients [13]. Therefore, we computed the IC_50_ values for several chemotherapeutic drugs (Cisplatin, Niraparib, Olaparib, and Paclitaxel) using the oncoPredict algorithm and compared the results across the two categories of risk in the TCGA cohort. As shown in Figure 6A, all of these medications' estimated IC_50_ values were higher in OC patients with a high-risk score, of which the estimated IC_50_ values of Niraparib and Olaparib were significantly higher, implying that individuals with a low-risk score may be more sensitive to these drugs. Furthermore, we applied our model to two actual chemotherapy cohorts to investigate its potential for chemotherapeutic benefit prediction. In the GSE30161 cohort (Figure 6B), patients with complete response (CR) to carboplatin/taxol/cisplatin/cytoxan therapy had a significantly lower stemness-risk score than patients with partial response (PR), and individuals in the low-risk cohort underwent superior therapeutic outcomes compared to those in the high-risk cohort (59.5% versus 55.6%; Figure 6C). Similarly, in Cancer Center cohort [14], patients who are resistant to carboplatin/taxol/cisplatin therapy had higher stemness-risk scores (Figure 6D), and in the low stemness-risk group, the frequency of sensitive was likewise significantly higher (66.6% versus 36.4%; Figure 6E). These results suggested that chemotherapy may be sensitive for OC patients with a low stemness-risk score.

### 3.6. Clinical Validation of the Signature for Chemotherapeutic Response

**Figure 7:**
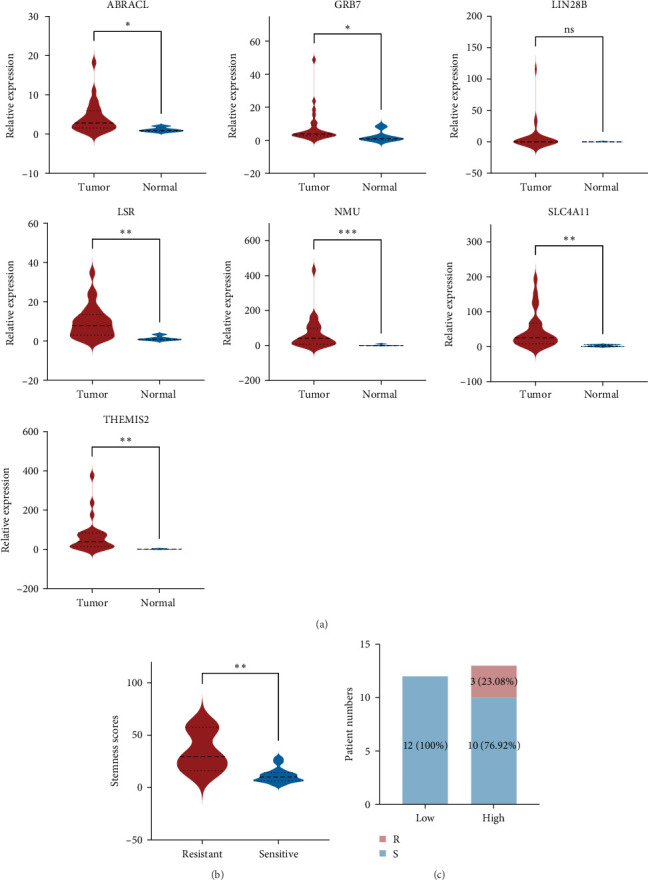
Results of real time-PCR experiments. (A) Relative mRNA expression of seven-screened genes in five normal tissues and 25 ovarian cancer (OC) samples derived from cancer center. (B) Stemness scores of patients between chemoresistant and chemosensitive groups in Cancer Center cohort. (C) Distributions of responder (S) and nonresponder (R) to chemotherapy in high- and low-risk group from cancer center.

We also used a PCR array to measure the expression level of the selected seven SRGs in a clinical cohort of 25 OC patients with a median age of 52.72 years (range 27–67 years) in order to investigate the viability of converting our signature into a clinical risk stratification assay. The study cohort comprised 25 high-grade serous adenocarcinomas (HGSOC). Comparative analysis revealed elevated mRNA expression levels of all seven SRGs in tumor tissues compared to corresponding normal tissue of five patients (Figure 7A). Obviously, patients who were sensitive to chemotherapy had risk scores that were noticeably lower than those of patients who were resistant to chemotherapy (Figure 7B). Additionally, patients were divided into low-risk and high-risk groups based on the median risk score (11.26), which was used as the cut-off point. Individuals in the low-risk cohort benefited more from therapy than those in the high-risk cohort (100% versus 76.92%; Figure 7C).

### 3.7. THEMIS2 Knockdown Diminishes CSC Features of OC Cells

**Figure 8:**
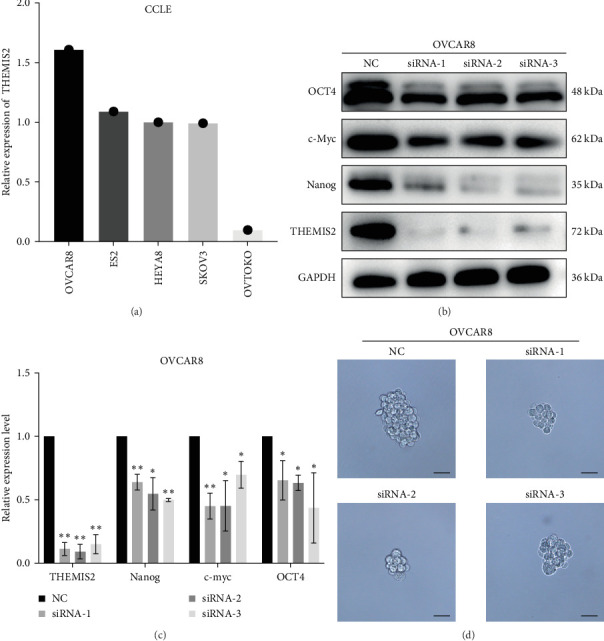
Results of experiments. (A) Relative mRNA expression of thymocyte selection associated family member 2 (THEMIS2) in five ovarian cancer (OC) cell lines from CCLE. (B) Western blot showed the expression of stem-related markers OCT4, c-Myc, and Nanog was downregulated in si-THEMIS2 transfected OVCAR8 cells. (C) The bar graph represents relative protein gray value of THEMIS2, OCT4, c-Myc, and Nanog after THEMIS2 deletion. (D) Representative images of OVCAR8 cell spheres after transfection with NC and si-THEMIS2-1, -2 and -3 for 7 days, respectively. Scale bar, 50 μm.

We functionally confirmed THEMIS2's putative role in promoting CSC properties by using random forest survival analysis to screen out of the seven genes in the stemness signature as the most significant gene (Figure S1D). As shown in Figure 8A, the gene expression of cell lines acquired from Demap revealed that the mRNA level of THEMIS2 was higher in the OVCAR8 cell line than in the others, and the expression of THEMIS2 in OVCAR8 cells was effectively inhibited by the use of particular siRNAs (Figure 8B,C). Furthermore, we used western blot to analyze the stem cell markers c-Myc, Nanog, and OCT4 protein expression. The results showed that si-THEMIS2 transfection reduced c-Myc, Nanog, and OCT protein levels, suggesting that THEMIS2 impacted the CSC characteristics of OC cells (Figure 8B,C). In addition, the sphere formation assay was used to evaluate the stemness characteristics of THEMIS2. The findings showed that si-THEMIS2 transfection significantly reduced the number and size of spheres in OVCAR8 cells and suggested that stemness-exhibiting OC cells were suppressed (Figure 8D).

## 4. Discussion

OC, considered one of the most severe gynecological tumors, is characterized by a high degree of heterogeneity, leading to significant variability in individual outcomes. Currently, treatment modalities for OC mainly comprise a combination of surgical debulking along with chemotherapy or radiotherapy. Nevertheless, the prognosis for OC remains suboptimal, primarily due to late-stage diagnosis, drug resistance, and recurrence. According to previous research [15], the identification of a reliable signature for evaluating patient outcomes and guiding therapeutic strategies would significantly improve the treatment and prognosis of OC.

CSCs, owing to their phenotypic plasticity and dynamic equilibrium with complex intrinsic/extrinsic microenvironmental niches, exert indispensable roles in OC development and persistence. Stemness-acquired OC cells have been shown to survive conventional chemotherapy [16] and drive peritoneal and omental metastasis [17], implicating stemness in OC recurrence. Comparative analyses reveal that cisplatin-resistant, metastatic, and recurrent OC cells exhibit elevated stemness-associated traits compared to their counterparts, while chemotherapy exposure (cisplatin or paclitaxel) further potentiates stemness acquisition in OC cell populations [18, 19]. Accumulating evidence indicates that multiple CSC markers (e.g., ALDH and c-kit/CD117) and conserved signaling pathways (e.g., Wnt and Notch) are critically involved in OC pathogenesis, including tumor initiation, transcoelomic metastasis, hematogenous dissemination, and chemoresistance [20]. These findings highlight the translational potential of CSC-directed therapeutic strategies for achieving durable remission in OC patients. Therefore, it seems that the systematic integration of bioinformatics-driven tumor stemness evaluation presents a clinically viable approach for predicting therapeutic sensitivity and optimizing personalized treatment strategies in OC patients. Interestingly, our findings indicate that the heterogeneous stemness profiles enable stratification of OC patients into distinct prognosis and therapeutic response groups, providing a rationale for stemness-informed therapeutic decision-making.

In the present study, a comparison was made between the expression patterns of SRGs in ovarian tissue and OC tissue. Then, stemness-relevant prognostic DEGs were identified by the univariate Cox regression analysis. Furthermore, a random forest survival analysis was implemented to screen significant genes associated with the prognosis. From 127 combinations of seven genes, we constructed a stemness-relevant prognostic gene signature consisting of seven genes (ABRACL, GRB7, LIN28B, LSR, NMU, SLC4A11, and THEMIS2) to quantify the stemness pattern. Using the signature formula, OC patients were stratified into low- and high-stemness risk groups. The Kaplan–Meier and ROC curve analysis collectively revealed that individuals in the high-risk group demonstrated significantly shorter OS times. Regarding chemotherapy guidance, oncoPredict analysis suggested that a higher stemness-risk score was associated with higher IC_50_ values of chemotherapeutic drugs for OC patients. Interestingly, when applying our model to the GSE30161 and cancer center trials, consistent results were observed: low stemness-risk patients experienced more significant clinical benefits following carboplatin/taxol/cisplatin therapies, thus, affirming the predictive validity of our stemness model.

In relation to TME patterns, several studies have demonstrated a close association between the OC stemness-risk and the tumor environment, including immune cell composition [21]. Mounting evidence has underscored the complex interaction between CSCs and the tumor microenvironment, which can drive cancer progression [22]. In gastric cancer, mesenchymal stem cells stimulated by activated CD4+ T cells, have been shown to enhance the migration and growth potential of gastric cancer cells in BALB/c nu/nu xenografts [23]. In addition, tumor-educated neutrophils can activate mesenchymal stem cells to facilitate the growth and metastasis of gastric cancer [24]. In OC, M2 macrophages have been found to increase the proliferation of sphere cells derived from OC stem-like cells [25]. The results from the TIMER2.0 indicated that a high stemness-risk score was related to a high abundance of CD4+ T cells, macrophages, and neutrophils, while a low stemness-risk score was correlated with CD8+ T cells enrichment. The precise functions of these cells in OC stemness cells need further investigation. On the contrary, CSCs, which serve as the driving force behind tumor development, initiate the generation of new cells by modulating various signaling pathways [26]. In this context, the gene set variation analysis (GSVA) found that high stemness-risk group exhibited enrichment in pathways related to the mitotic spindle, glycolysis, G2/M checkpoint, E2F targets, and the PI3K/AKT/mTOR signaling pathway.

For the seven stemness model genes identified in this study, ABRACL has been proposed as a prospective biomarker in endometrial cancer uterine aspirate [27], and Li and Chen [28] reported that ABRACL deletion could suppress proliferation, invasion, migration, and EMT of breast cancer cells. GRB7 can regulate angiogenesis via VEGFA/VEGFR2 signaling as well as its downstream pathways in OC [29]. Pei et al. [30] reported that gastric cancer cells with elevated expression of GRB7 showed a positive correlation with the self-renewal capability of gastric CSCs. Ring finger protein 144A (RNF144A) [31] and circular nuclear factor IX (circNFIX) [32] contribute to the stemness properties of OC cells through LIN28B. High levels of LSR, and NMU were associated with poor prognosis in OC. The suppression of THEMIS2 promotes the proliferation of both BC cells [33] and OC cells [34] in vitro, respectively. However, THEMIS2 is upregulated in the CSCs of the TNBC and OC cell lines, and it augments cancer stemness and chemoresistance by releasing PTP1B from MET in TNBC [35]. In this study, we revealed that the mRNA levels of seven stemness model genes were higher in OC tissues than in normal tissues using a PCR array from our center. The overexpression of these genes might be a hint that these OC patients would experience chemotherapy resistance and have a poor prognosis. Elucidating the biochemical mechanisms of these genes in the progression of OC and expecting to find new targets in the treatment of OC will be of great significance to improve patient quality of life and prolong their survival. Furthermore, we have found that THEMIS2 knockdown could inhibit the induction of differentiation-associated genes and attenuate the number and size of the spheres, supporting critical roles of THEMIS2 in CSCs of OC cell lines. Following, we will shed light upon the underlying mechanism to elucidate the specific way that THEMIS2 affects the CSCs of OC cell lines and evaluate the feasibility of THEMIS2 as a novel molecular target of therapy for OC.

## 5. Conclusion

Taken together, through applying univariate Cox regression analysis and random survival forest analysis, we developed and validated a seven stemness-related prognostic gene signature for OC, drawing from independent cohorts. The signature has prospective clinical application for prognosis evaluation. Furthermore, we propose that our stemness model could assist physicians in identifying prospective responders, allowing the preferential use of existing chemotherapeutic drugs.

## Figures and Tables

**Table 1:** Correlation between risk score and clinicopathologic characteristics of ovarian cancer (OC) patients in Cancer Center cohort.

Characteristics	Cancer Center cohort		*p* Value^a^
	High risk (*n* = 13)	Low risk (*n* = 12)	—
Age (year)			>0.999
≤50	5 (38.5%)	4 (33.3%)	—
>50	8 (61.5%)	8(66.7%)	—
Type			
HGSC	13 (100%)	12(100%)	—
Grade			0.9104
G1–G2	3 (23.1%)	3 (25.0%)	—
G3	10 (76.9%)	9 (75.0%)	—
TNM stage			>0.999
Stage I–II	5 (38.5%)	5 (41.7%)	—
Stage III–IV	8 (61.5%)	7(58.3%)	—

^a^The constituent ratio of each feature between high risk subtype and low risk subtype was compared using the Pearson chi-square test.

**Table 2:** Clinicopathologic characteristics of OC patients in training and validation cohort.

Characteristics	TCGA cohort	GSE30161 cohort
Samples size	419	58
Median OS (day)	1003 (8–5481)	1375 (49–4208)
Number of deaths	233 (55.6%)	36 (62.1%)
Age (year)		
≤50	101 (24.1%)	7 (12.1%)
>50	318 (75.9%)	51 (87.9%)
Histologic type		
Serous	419 (100%)	47 (81.0%)
Clear cell	0	5 (8.6%)
Endometrioid	0	1 (1.7%)
Mucinous	0	1 (1.7%)
Transitional cell	0	1 (1.7%)
Undifferentiated	0	1 (1.7%)
Histologic grade		
G1–G2	48 (11.5%)	—
G3	360 (85.9%)	—
Stage		
I–II	26 (6.2%)	0
III–IV	390 (93.1%)	58 (100%)
Grade		
Poor	—	33 (56.9%)
Mod	—	19 (32.8%)
Well	—	2 (3.4%)

Abbreviations: OC, ovarian cancer; OS, overall survival; TCGA, The Cancer Genome Atlas.

## Data Availability

The data used to support the findings of this study are available from the corresponding author upon request.

## Supporting Information

Supporting Information The supporting information include the sequences of primers and some statistical analysis results. Figure S1 include stemness-related DEGs from TCGA and GTEx, variable importance of 14 genes, and top 10 gene signatures. Figure S2 shows the relative expression of seven-selected genes in normal tissue in GTEx and OC samples in the TCGA dataset. Figure S3 shows correlation of seven-selected genes with immune cells in OC. Table S1 shows 14 candidate stemness-related genes identified by univariate Cox regression analysis. Tables S2 and S3 include correlation of risk score with clinicopathologic characteristics of OC patients in the TCGA cohort and GSE30161 cohort, respectively. Table S4 lists sequences of primers used for real-time PCR.
