# Supplementary material for: Stemness-Relevant Gene Signature for Chemotherapeutic Response and Prognosis Prediction in Ovarian Cancer
Source: Stem Cells Int. 2025 Mar 27;2025:2505812. doi: 10.1155/sci/2505812 (PMC11968171; doi:10.1155/sci/2505812)
Supplement: Supporting Information — The supporting information include the sequences of primers and some statistical analysis results. Figure S1 include stemness-related DEGs from TCGA and GTEx, variable importance of 14 genes, and top 10 gene signatures. Figure S2 shows the relative expression of seven-selected genes in normal tissue in GTEx and OC samples in the TCGA dataset. Figure S3 shows correlation of seven-selected genes with immune cells in OC. Table S1 shows 14 candidate stemness-related genes identified by univariate Cox regression analysis. Tables S2 and S3 include correlation of risk score with clinicopathologic characteristics of OC patients in the TCGA cohort and GSE30161 cohort, respectively. Table S4 lists sequences of primers used for real-time PCR. [file 2505812.f1.docx]

## Supplementary Materials

## Supplementary Figures and Tables

## Supplementary Figures


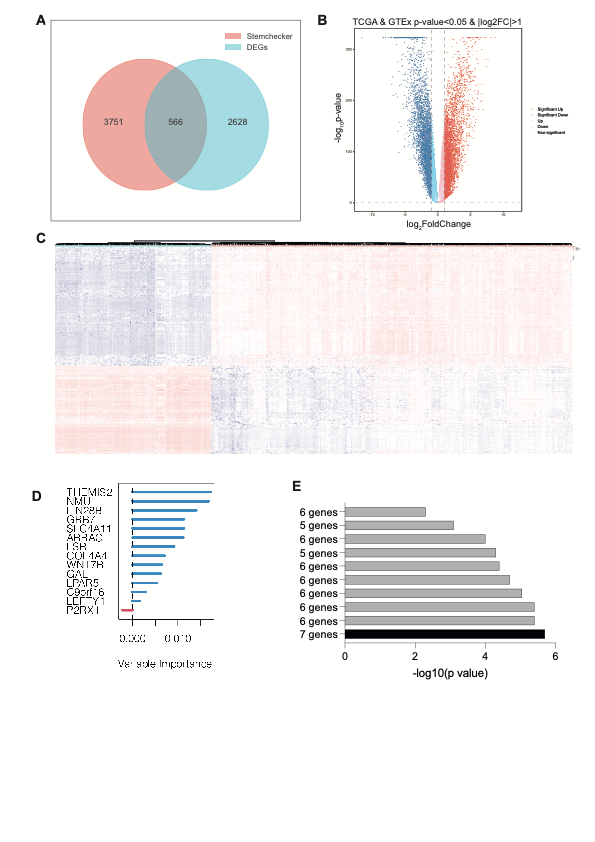


**Supplementary Figure 1**. (A) Venn Diagram shows stemness-related genes from stemChecker database and DEGs between TCGA and GTEx. (B) Volcano plot showed fold changes (x-axis) and corresponding P values (y-axis) of DEGs between GTEx and OC samples in the TCGA dataset. (C) Heatmap manifesting the expression of selected 566 stemness-related DEGs from TCGA and GTEx. (D) Variable importance of 14 genes calculated by random survival forest algorithm. (E) Top 10 gene signatures from 127 (27-1) gene alignment assemblies evaluated by log-rank tests, which were displayed as the -log10(log-rank P) values, and the 7-gene signature marked in black was chosen as the final model.

**Supplementary Figure 2**. The relative expression of 7-selected genes in normal tissue in GTEx and OC samples in the TCGA dataset.


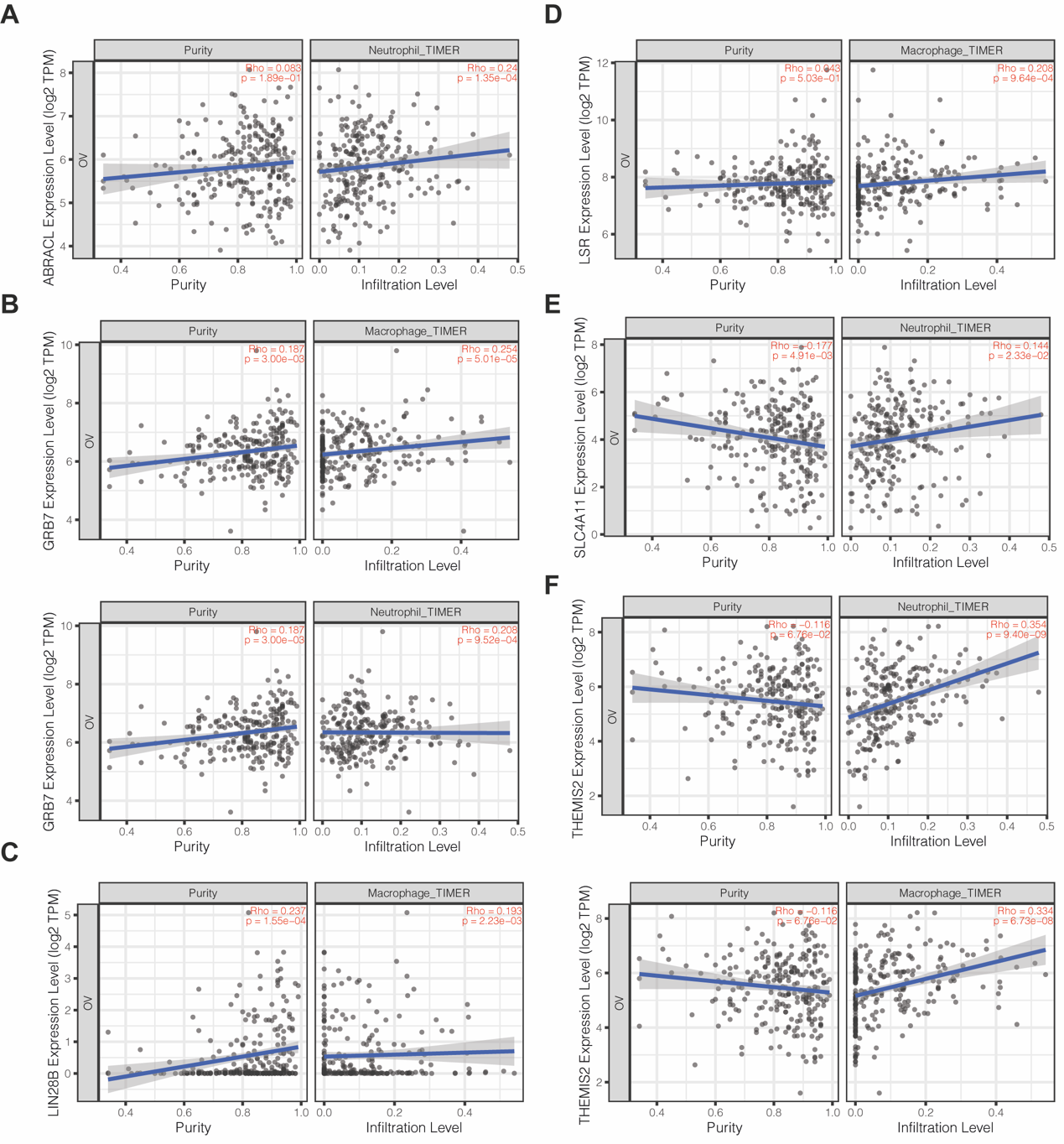


**Supplementary Figure 3**. Correlation with immune cells in OC. (A) The relationship between neutrophils and ABRACL. (B) The relationship between neutrophils, macrophages and GRB7. (C) The relationship between macrophages and LIN28B. (D) The relationship between macrophages and LSR. (E) The relationship between neutrophils and SLC4A11. (F) The relationship between neutrophils, macrophages and THEMIS2.

## Supplementary Tables

**Supplementary Table 1.** Fourteen candidate stemness-related genes identified by univariate Cox regression analysis

| Gene | Hazard ratio | 95% confidence interval | *P* value |
| --- | --- | --- | --- |
| ABRACL | 1.211 | 1.021- 1.437 | 0.027 |
| C9orf16 | 1.224 | 1.013- 1.479 | 0.036 |
| COL4A4 | 0.815 | 0.640- 1.037 | 0.097 |
| GAL | 1.123 | 1.034-1.220 | 0.005 |
| GRB7 | 1.174 | 1.022-1.349 | 0.023 |
| LEFTY1 | 0.845 | 0.737-0.968 | 0.015 |
| LIN28B | 1.111 | 0.984-1.255 | 0.086 |
| LPAR5 | 1.144 | 0.996-1.314 | 0.055 |
| LSR | 1.201 | 1.008-1.430 | 0.040 |
| NMU | 1.100 | 1.001-1.208 | 0.046 |
| P2RX1 | 0.797 | 0.643-0.987 | 0.037 |
| SLC4A11 | 1.098 | 1.004-1.201 | 0.0394 |
| THEMIS2 | 1.102 | 0.984-1.235 | 0.091 |
| WNT7B | 1.120 | 1.014-1.237 | 0.024 |

**Supplementary Table 2.** Correlation of risk score with clinicopathologic characteristics of OC patients in the TCGA cohort

| Characteristics | High Risk  (n = 210) ^a^ | Low Risk  (n = 209) ^a^ | *P* value^b^ |
| --- | --- | --- | --- |
| Age (year) |  |  | 0.7112 |
| ≤ 50 | 49(23.3%) | 52 (24.9%) |  |
| > 50 | 161(76.7%) | 157 (75.1%) |  |
| Histologic grade |  |  | 0.3201 |
| G1- G2  G3 | 27 (13.4%)  175(86.6%) | 21 (10.2%)  185(89.8%) |  |
| Stage  I-II  III-IV | 13(6.25%)  195(93.75%) | 10(9.52%)  195(90.48%) | 0.5433 |
| Venous invasion |  |  | 0.8696 |
| YES | 32 (58.2%) | 34 (56.7%) |  |
| NO | 23 (41.8%) | 26 (43.3%) |  |
| Lymphatic invasion |  |  | 0.5907 |
| YES | 54 (66.7%) | 52 (62.7%) |  |
| NO | 27 (33.3%) | 31 (37.3%) |  |
| ^a^ Sum of the frequency of some variables may not be equal to the total sample size due to the missing values;  ^b^ The constituent ratio of each feature between proliferation subtype and non-proliferation subtype was compared using the Pearson chi-square test. | | | |

**Supplementary Table 3.** Correlation of risk score with clinicopathologic characteristics of OC patients in GSE30161 cohort

| Characteristics | GSE30161 cohort | | *P* value ^a^ |
| --- | --- | --- | --- |
|  | High risk  (n = 29) | Low risk  (n = 29) |  |
| Age (year) |  |  | >0.999 |
| ≤ 50 | 3 (9.4%) | 3 (9.4%) |  |
| > 50 | 26 (90.6%) | 26(90.6%) |  |
| Grade |  |  | 0.0507 |
| Well-Mod | 7 (25.9%) | 14 (51.8%) |  |
| Poor | 20 (74.1%) | 13 (48.2%) |  |
| TNM stage |  |  | 0.3683 |
| Stage III a- III b | 6 (53.7%) | 9(69.7%) |  |
| Stage IIIc-IV | 23 (46.3%) | 20 (30.3%) |  |

^a^ The constituent ratio of each feature between proliferation subtype and non-proliferation subtype was compared using the Pearson chi-square test.

**Supplementary Table 4**．Sequences of primers used for real-time PCR.

| **Primer** | **Sequence 5′-3′** |
| --- | --- |
| GAPDH forward | CTGGGCTACACTGAGCACC |
| GAPDH reverse | AAGTGGTCGTTGAGGGCAATG |
| ABRACL forward | ACCTCTTTGAAGCATTGGTAGG |
| ABRACL reverse | GCAGCTCTCCTGGATATGTTAC |
| LIN28B forward | TTGAGTCAATACGGGTAACAGGA |
| LIN28B reverse | TGACAGTAATGGCACTTCTTTGG |
| GRB7 forward | CGGAAAAACTTCGCCAAGTACG |
| GRB7 reverse | CCAGTGTGTGCATCGAGACAG |
| NMU forward | CTCAGGCATCCAACGCACT |
| NMU reverse | GACTTGCCCAACTTCTGTGTC |
| LSR forward | TACAACCCCTACGTTGAGTGC |
| LSR reverse | CCTCCGGCCCTGGTAGTAAT |
| SLC4A11 forward | CCTCCCGAAAGTACCTGAAGT |
| SLC4A11 reverse | CCAGGAAGCCATCTAGGTCG |
| THEMIS2 forward | CTCAAGTCTTAGGATTGCAGCA |
| THEMIS2 reverse | CTGTGGGCAGGAATCTTGC |
